# Supplementary material for: Functional Diversity of the Litter-Associated Fungi from an Oxalate-Carbonate Pathway Ecosystem in Madagascar
Source: Microorganisms. 2021 May 1;9(5):985. doi: 10.3390/microorganisms9050985 (PMC8147286; doi:10.3390/microorganisms9050985)

Phylogenetic tree of the *Ceratobasidiaceae* (Basidiomycota) with *Trechispora* as outgroup

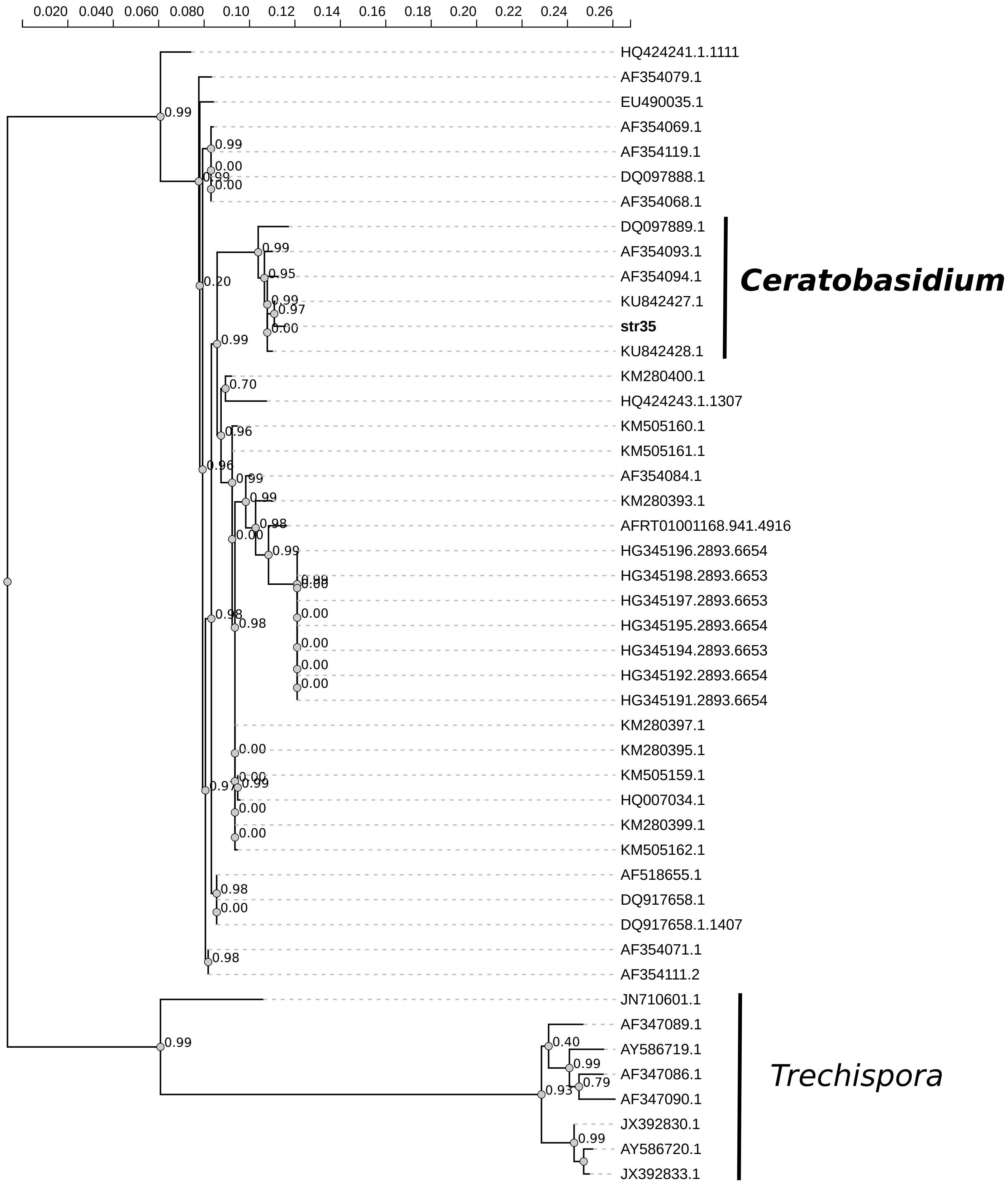

Phylogenetic tree of the *Mucorales* (Mucoromycota) with *Umbelopsis* as outgroup

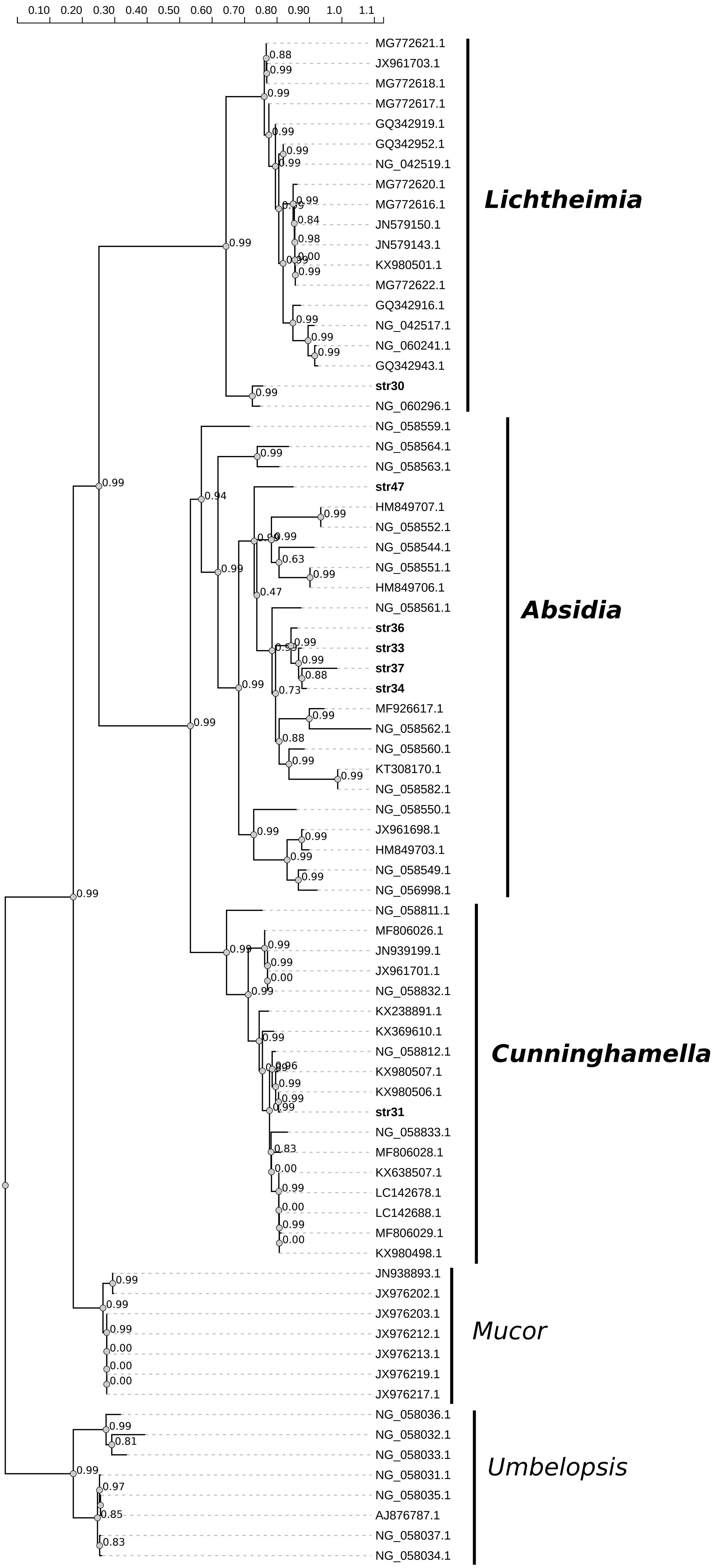

Phylogenetic tree of the *Dothideomycetes* (Ascomycota) with *Saccharomyces* as outgroup

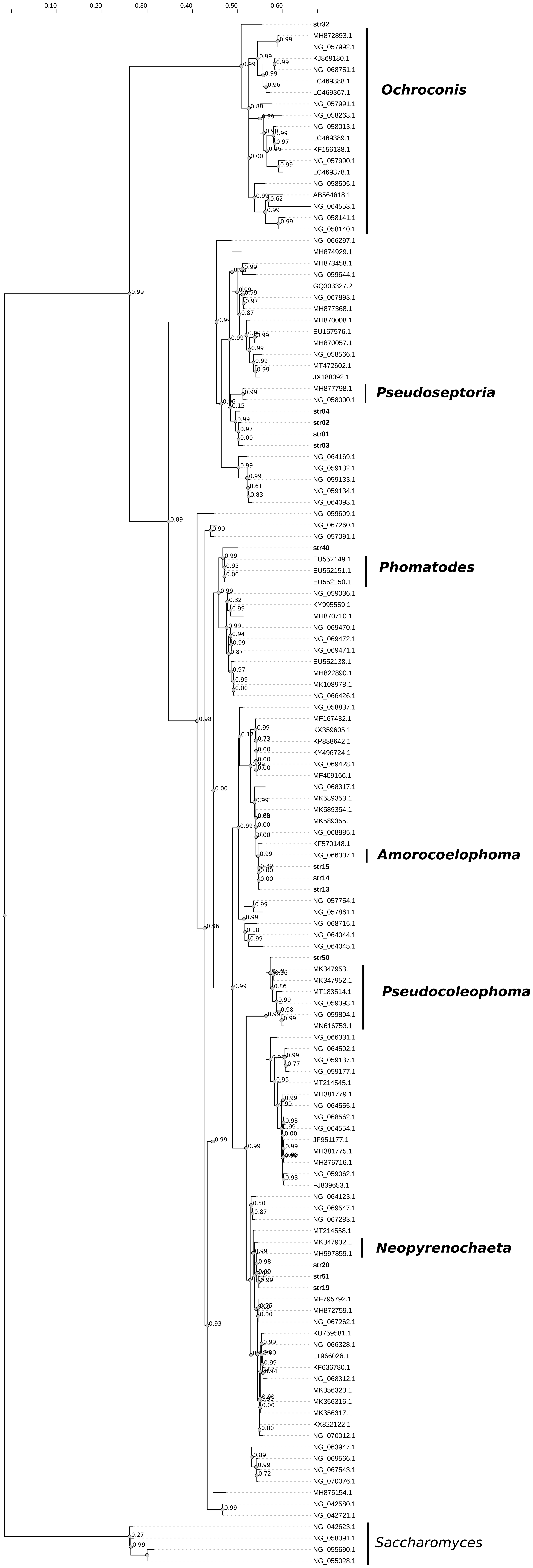

Phylogenetic tree of the *Eurotiomycetes* (Ascomycota) with *Saccharomyces* as outgroup

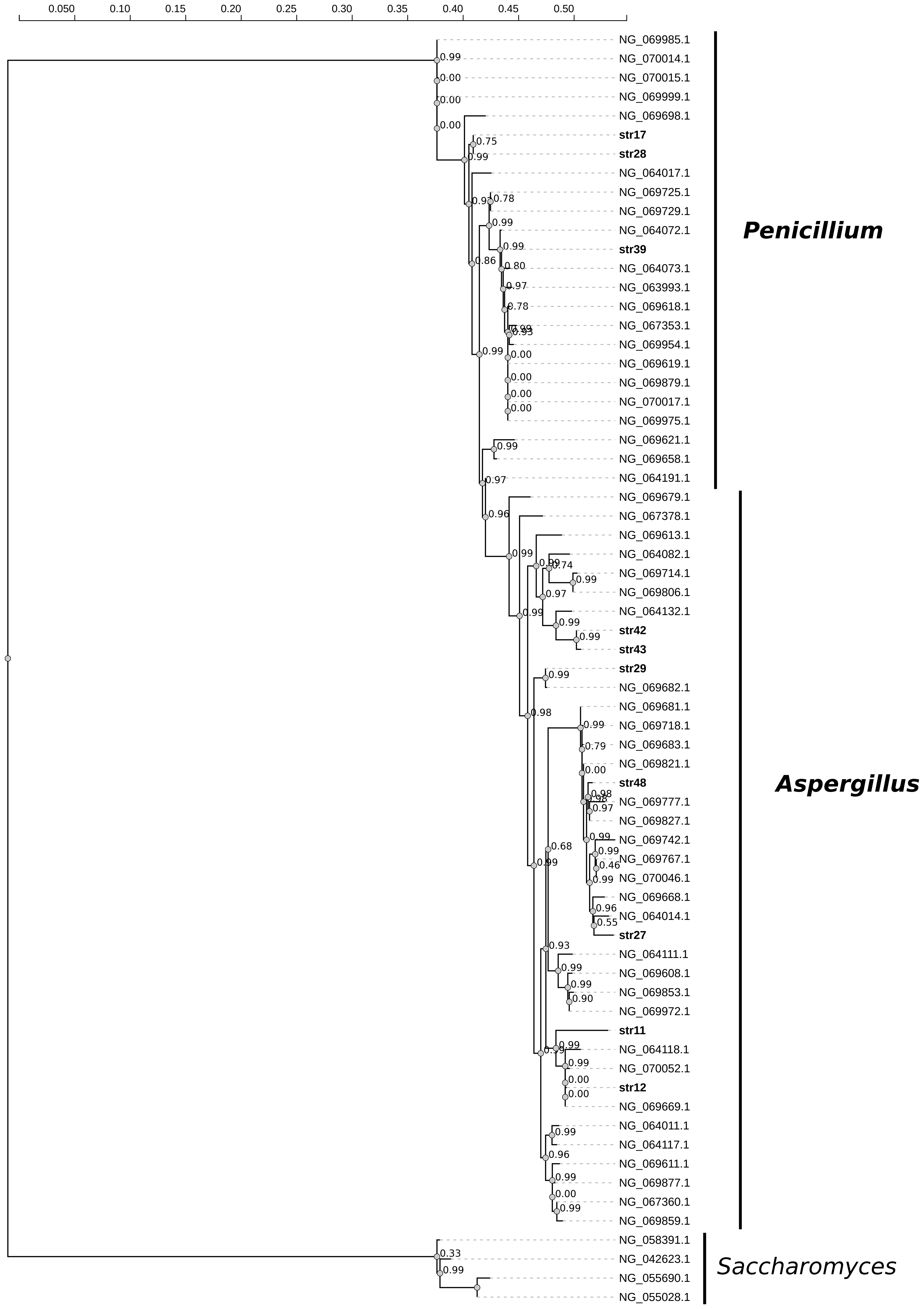

Phylogenetic tree of the *Leotiomycetes* (Ascomycota) with *Saccharomyces* as outgroup

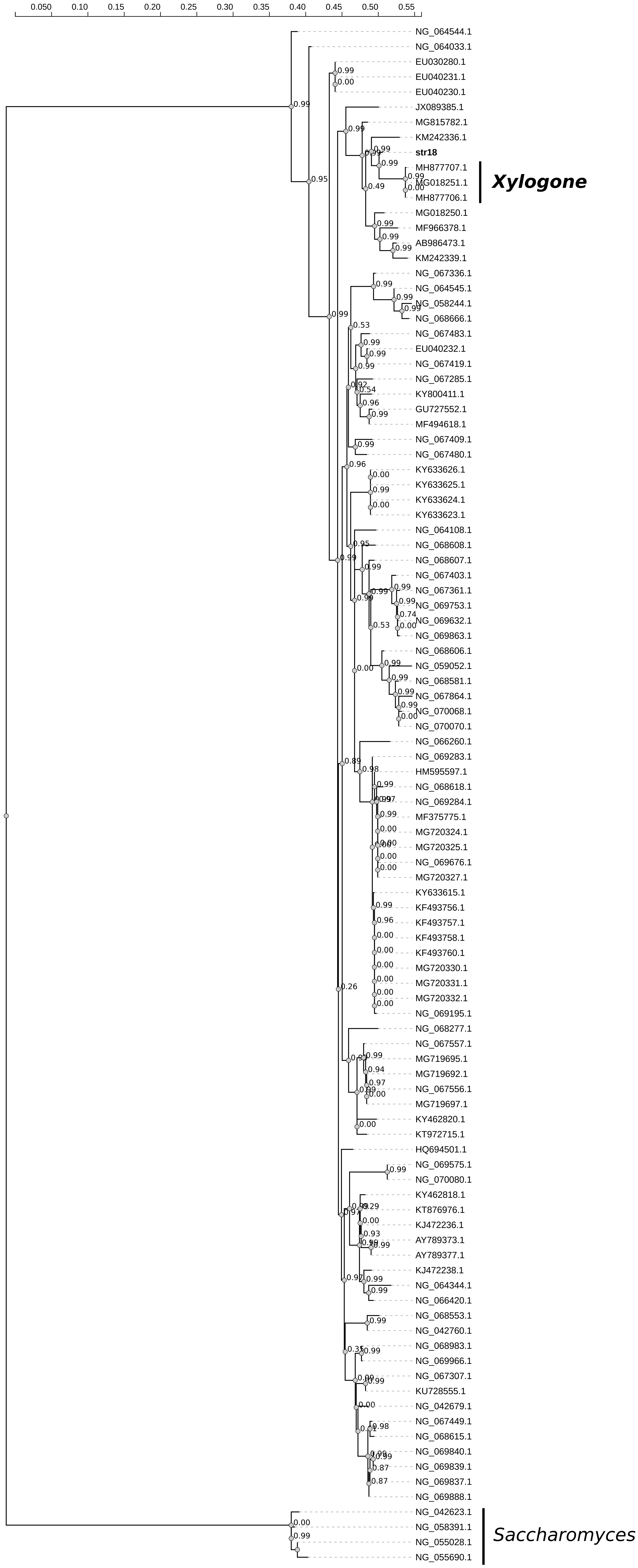

Phylogenetic tree of the *Sordariomycetes* (Ascomycota) with *Saccharomyces* as outgroup

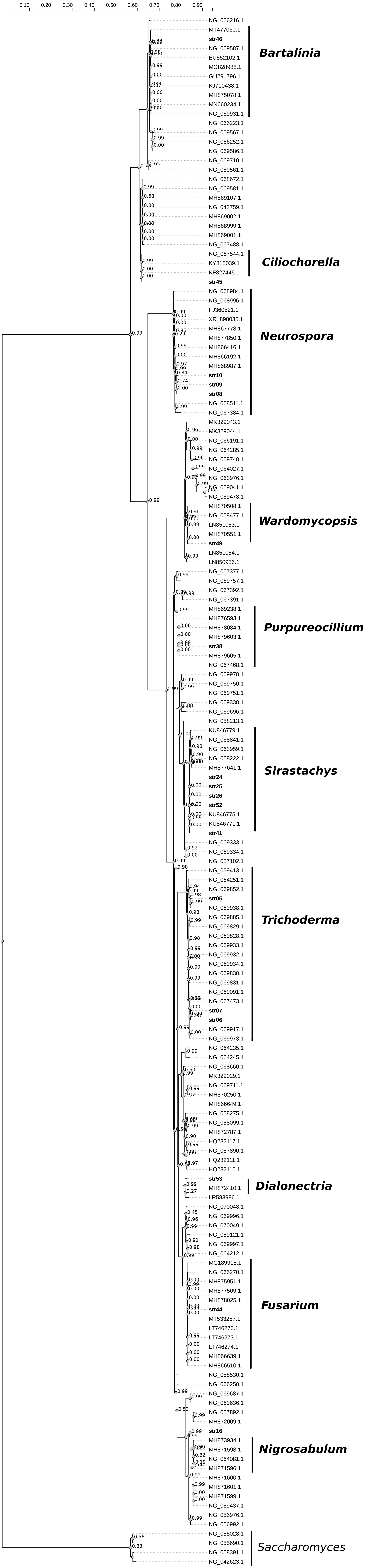

Supplement: Supplementary file 1 [file microorganisms-09-00985-s001.zip › Figure_S1.pdf]
